# Improvement of the Physical Properties of Guided Bone Regeneration Membrane from Porcine Pericardium by Polyphenols-Rich Pomace Extract

**DOI:** 10.3390/ma12162564

**Published:** 2019-08-12

**Authors:** Nazario Russo, Clara Cassinelli, Elisa Torre, Marco Morra, Giorgio Iviglia

**Affiliations:** 1 Specialization School EIMS-UFP, University of Cagliari, Via Università 40, 09124 Cagliari (CA), Italy; 2 Nobil Bio Ricerche srl, Via Valcastellana 26, 14037 Portacomaro (AT), Italy

**Keywords:** guided bone regeneration, membrane, porcine pericardium, biomaterial, polyphenols, antioxidant

## Abstract

To achieve optimal performances, guided bone regeneration membranes should have several properties, in particular, proper stiffness and tear resistance for space maintenance, appropriate resorption time, and non-cytotoxic effect. In this work, polyphenol-rich pomace extract (PRPE), from a selected grape variety (Nebbiolo), rich in proanthocyanidins and flavonols (e.g., quercetin), was used as a rich source of polyphenols, natural collagen crosslinkers, to improve the physical properties of the porcine pericardium membrane. The incorporation of polyphenols in the collagen network of the membrane was clearly identified by infra-red spectroscopy through the presence of a specific peak between 1360–1380 cm^−1^. Polyphenols incorporated into the pericardium membrane bind to collagen with high affinity and reduce enzymatic degradation by 20% compared to the native pericardium. The release study shows a release of active molecules from the membrane, suggesting a possible use in patients affected by periodontitis, considering the role of polyphenols in the control of this pathology. Mechanical stiffness is increased making the membrane easier to handle. Young’s modulus of pericardium treated with PRPE was three-fold higher than the one measured on native pericardium. Tear and suture retention strength measurement suggest favorable properties in the light of clinical practice requirements.

## 1. Introduction

Traditional periodontal surgery techniques, such as gingivectomy, open flap debridement, and osseous surgery [1,2,3,4], allow the specialist to remove plaque, calculus, and endotoxins [5,6,7] in diseased tissues. Among the several oral conditions, periodontal disease is the most prevalent [8], causing tooth loss following inflammation and destruction of tissues in the periodontal site [6,9,10,11]. Titanium dental implants are the common surgical treatment of choice for replacing missing teeth [12,13]. The surgical feasibility is mainly dependent on the quality and quantity of bone in the periodontal site; in fact, the activation of specific cytokines in the context of a chronic inflammatory process leads to bone resorption which represents the most critical factor in determining an implant’s stability. Thanks to advancements in the field of dentoalveolar reconstruction, regeneration of new alveolar bone is made increasingly possible thanks to the use of so-called bone grafting materials, either autologous, or from an animal source or synthetic [14,15], which allow the formation of long junctional epithelium and new connective tissue in the periodontal wound [16]. The predictable tissue integration of dental implants is essential to restore the correct functions in patients. For a successful regeneration of periodontal tissues, much consideration is given to guided tissue regeneration (GTR), a treatment modality proposed by Melcher [17] based on the tissue compartment hypothesis, in which the selective growth of cells derived from the periodontal ligament is achieved through placing a physical barrier, in order to prevent the migration of epithelial and gingival connective tissue cells along the root surface. Specifically, because of the different growth potential of periodontal cells, the type of cell which repopulates the root surface determines the nature of the attachment and, in case of a migration of epithelial and fibroblast cells, inhibition of bone formation can occur [16,18]. Karring et al. were the first to experimentally and histologically demonstrate Melcher’s hypothesis [17,19], having achieved bone regeneration by excluding epithelial cells and fibroblasts from the wound space and, thus, allowing pre-osteoblast cells to migrate in the bone graft and to produce the mineralized matrix required for bone tissue regeneration. The first material used for GTR was a cellulose acetate laboratory filter, and it was the first time when periodontal regeneration was achieved using the GTR technique [20]. From that moment, various types of membranes with different properties, such as tissue integration, cell occlusivity, space-making ability, and biocompatibility, have been developed [21,22,23,24] and are able to create a space to protect the bone graft in order to preferentially allow bone growth into the space. Furthermore, GTR can be performed with different kinds of materials to be employed in membrane manufacturing, thus creating both resorbable and non-resorbable membranes. Non-resorbable membranes, most of which are made of expanded polytetrafluoroethylene (ePTFE) material, have the disadvantage that they need to be removed in a second surgery procedure, but have the advantage in that they do not generate antigenicity [25]. Resorbable membranes, on the contrary, do not need any additional surgery procedure to be removed, because they are made of natural materials which are biocompatible and undergo progressive degradation during tissue formation. The most used material for resorbable membranes is collagen from bovine or porcine sources [26,27], however, because bacteria responsible for periodontitis are able to stimulate the production of collagenase enzymes, the degradation process of the membranes could be accelerated, thus making the regeneration unpredictable [28,29,30]. To overcome this un-controlled degradation rate, some clinically available collagen membranes are crosslinked using chemical compounds such as glutaraldehyde, with the disadvantage that they could cause cytotoxicity and an inflammation response in the surrounding tissue [31,32].

Increasing collagen GTR membranes’ durability without introducing side-effects is a goal of research in this area. Park and coworkers recently presented animal studies involving an 1-ethyl-3-(3-dimethylaminopropyl) carbodiimide (EDC) -cross-linked type-I collagen membrane [33]. A number of studies have been devoted to the evaluation of the effects on collagen structure and resistance to degradation of natural crosslinkers from plant sources, namely polyphenols (Pph) [34].

Pph are a class of chemical compounds that are mainly naturally biosynthesized by plants. They exert a number of actions in molecular defense mechanisms of the vegetable kingdom. They have a high affinity towards high proline proteins, such as collagen and elastin. The high density of polar phenolic hydroxyls contained in Pph molecules promotes collagen stabilization through hydrogen bonding; also, carbonyl groups contained in some classes of Pph interact through the formation of Schiff bases with collagen amino groups [34]. Among studies in this area, He and coworkers investigated collagen crosslinking promoted by procyanidin from grape seed extracts [35]. They demonstrated through differential scanning calorimetry and thermogravimetry that the collagen/procyanidin films have superior thermal stability in comparison with pure collagen, since the hydrogen bond interactions between collagen and procyanidin do not destroy the triple helix conformation of collagen. Choi and coworkers used proanthocyanidin as a crosslinking agent of collagen hydrogel, showing enhancement of the physical properties of the collagen scaffolds [36].

In addition to improving resistance to collagen degradation in biological systems, Pph have been found to stimulate bone formation, mineralization, as well as the proliferation, differentiation, and the survival of osteoblasts [37,38,39,40,41]. These effects are due to the stimulating effect of Pph on osteoblast cells and their antioxidant and anti-inflammatory effect [42]. In principle, the addition of Pph to collagen membranes could both improve stability to degradation and stimulate new bone formation, in the wake of present-day developments of GTR membranes [43].

A key issue in the exploitation of Pph properties is their source. The quoted studies on collagen stabilization by Pph involve either heterogeneous extracts or purified molecules. Polyphenol-rich pomace extract (PRPE) obtained through straightforward solid–liquid extraction [37,38,39] is of particular interest, because of availability, economical and ethical reasons. The main drawback is the complexity and the molecular heterogeneity of the mixture, and the high number of variables involved in the definition of extracts’ chemistry [40,41].

In this work we want to investigate PRPE from the Nebbiolo grapes’ pomace as a provider of polyphenols for enhancement of mechanical (through crosslinking) and biological (through release) properties of collagen membranes for GTR. The Nebbiolo grape was chosen among the many local varieties since it presented the highest phenolic content and antioxidant power. Starting from decellularized pericardium from the porcine source, we want to understand the effect of the complex polyphenols’ mixture obtained by solid–liquid extraction from pomace on the collagen structure; how it affects the resistance to degradation, both in normal fluid (PBS, phosphate buffered saline) and in collagenase solution; and how it affects mechanical properties and if it can provide the local release of polyphenols’ molecular species. Shortly, we will address the question of whether PRPE can introduce clinically significant advancements in GTR membranes.

## 2. Results

### 2.1. PRPE Characterization

The phenolic pattern of tested PRPE has been identified and quantified by different techniques. Analysis of the phenolic content shows an initial amount of 3.44 mg/mL of gallic acid equivalent (GAE) (Table 1). 2,2-Diphenyl-1-picrylhydrazyl (DPPH, Sigma-Aldrich, St. Louis, MO, USA) tests show that PRPE are endowed, as expected, by antioxidant properties and they reduce radicals in the DPPH solution by 44.3%. Among the Pph present in the tested PRPE, the test that involves the bleaching potential of sodium bisulfite, shows that 74.4 µg/mL of anthocyanins are contained in Nebbiolo PRPE.

**Figure 1:**
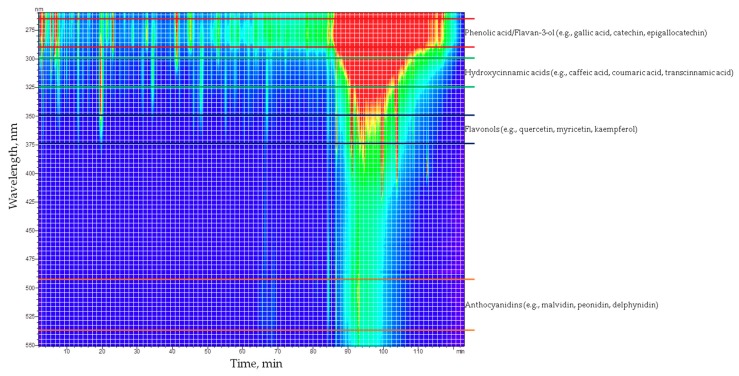
Spectrochromatogram of the PRPE from Nebbiolo’s grapes divided in sections corresponding to different polyphenol (Pph) classes.

The complex composition of the tested PRPE is well represented in Figure 1, which shows the spectrochromatogram of PRPE obtained by high performance liquid chromatography (HPLC) analysis. According to existing literature, preliminary qualitative analysis of the UV–Vis spectra of the peak found in the chromatograms allows the classification of the separated peaks in different classes: phenolic acid, hydroxycinnamic acid, flavonoids, flavonols, and anthocyanidins (Figure 1).

**Figure 2:**
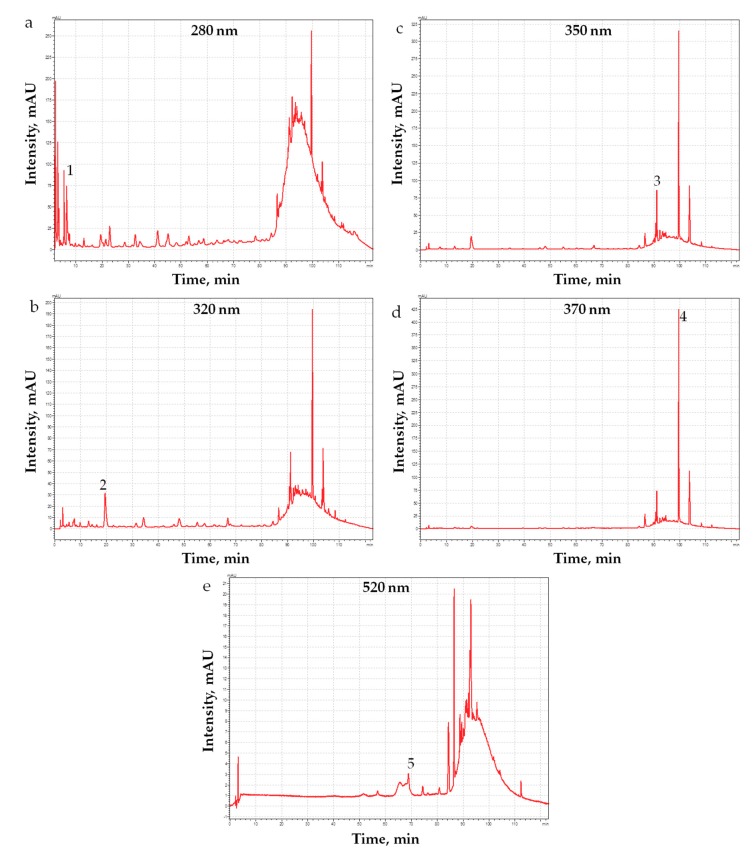
HPLC analysis of PRPE of Nebbiolo grape. Chromatograms at different wavelength: (**a**) 280, (**b**) 320, (**c**) 350, (**d**) 370, and (**e**) 520 nm are reported. Numbers indicate the peaks of the identified polyphenols: (1) gallic acid (280 nm), (2) caftaric acid, (3) rutin (350 nm), (4) quercetin (370 nm) and (5) malvidin-3-glucoside (520 nm) [45].

Phenolic acid and flavan-3-ol exhibit an absorbance maximum between 270 and 280 nm, hydroxycinnamic acid between 300–330 nm, sometimes with a shoulder around 290 nm, and flavonols in the range 350–375 nm and anthocyanidins show an absorbance maximum between 280–320 nm with a specific absorbance at 525 nm (Figure 1) [44]. In nature, polyphenols usually occur conjugated to sugars and organic acids, hence the comparison with the UV–Vis spectra and the retention time of standards do not allow a perfect identification of all compounds present in grape PRPE. However, it was possible to identify a few key components of PRPE, through the analysis of chromatograms at different wavelengths. As it is shown in Figure 2, well-resolved peaks that can be associated to phenolic acid and flavan-3-ol (flavonoids) are detected at 280 nm. Peaks at 320 nm are due to hydroxycinnamic acids, in particular, caftaric acid whose retention time is about 20 min [45]. Nebbiolo is a red grape, so it contains a multitude of anthocyanidins, such as malvidin, often found in the form of monoglucoside (anthocyanins), where the glucose molecule is linked through the hydroxyl group of anthocyanidins in position 3 (e.g., malvidin-3-glucoside). Furthermore, the peaks of rutin (350 nm) and quercetin (370 nm) are well-resolved and visible in the chromatograms in Figure 2. Apart from the qualitative detection of some Pph species, it was possible to quantify the following molecular species, by using four different standard solutions: quercetin, rutin, gallic acid, and malvidin-3-glucoside. Obtained results are shown in Table 2.

### 2.2. Polyphenols Content of Pericardium Treated with Polyphenols (P_PRPE)

**Figure 3:**
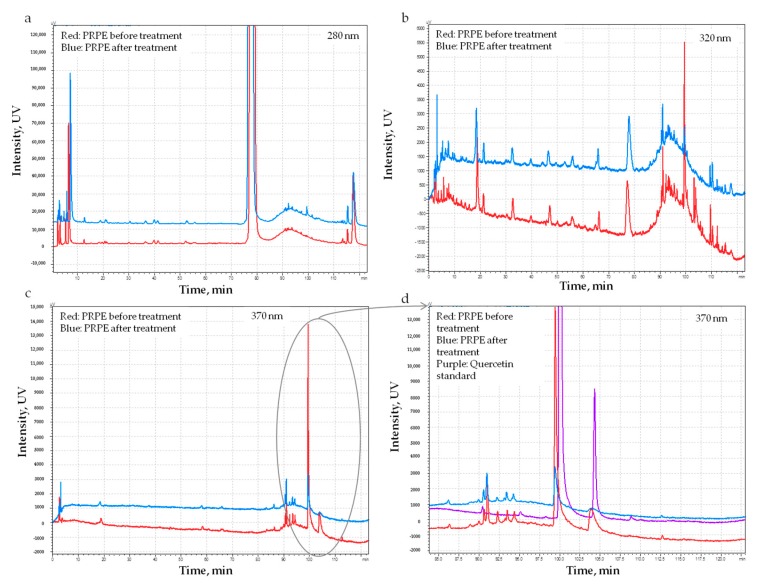
HPLC comparative analysis of PRPE before and after the treatment of the pericardium membrane at different wavelengths: 280 nm (**a**), 320 nm (**b**), and 370 nm (**c**). A marked decrease of quercetin peak has been reported after the treatment of the pericardium membrane (**d**).

According to the method described in Section 2.3, and in order to understand which kind of molecules and in which amount they were incorporated into the membrane during P_PRPE membrane preparation, the PRPE was analyzed before and after the treatment. In particular, Pph content of PRPE before soaking the membrane (50 mg of weight) was 3.4 mg and it decreased to 2.6 mg GAE after soaking; this means that around 0.8 mg of polyphenols have been incorporated in the pericardium fiber network. The antioxidant power of the PRPE before and after soaking also decreased coherently from 27.5% to 21.7%. It is noteworthy that chromatograms (Figure 3a–d) showed a marked decrease of the peak assigned to quercetin, at 370 nm, suggesting preferential incorporation of this molecule into the collagen network (Figure 3d).

### 2.3. Scanning Electron Microscopy

**Figure 4:**
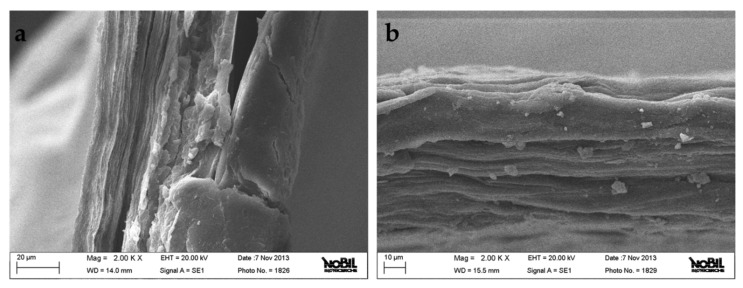
(**a**) SEM of pericardium control (P_CTRL). (**b**) SEM of pericardium treated with polyphenols (P_PRPE).

Observation of pericardium control (P_CTRL) and P_PRPE through SEM was performed to detect possible gross effects on morphology of the adopted crosslinking procedure. In general, no detectable differences were observed between the control and test sample. Qualitatively, sections of both samples show a layered structure (Figure 4a,b). Due to heterogeneity of the raw material, in terms of layer numbers and size, it was not possible to draw quantitative conclusions on, for example, possible effects of the preparation procedure on layer thickness.

### 2.4. Attenuated Total Reflectance Infrared Spectroscopy (ATR-IR)

**Figure 5:**
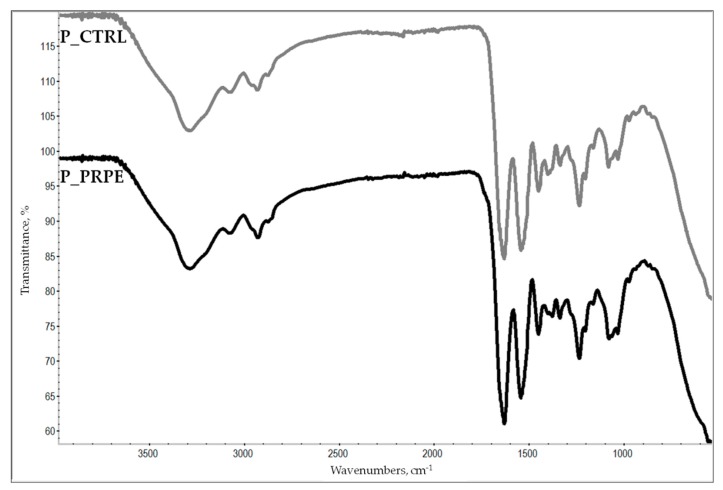
Attenuated total reflectance infrared spectroscopy (ATR-IR) of P_CTRL and P_PRPE between 4000 and 500 cm^−1^.

The ATR-IR spectra of P_CTRL and P_PRPE are shown in Figure 5.

Both spectra show the typical pattern of protein molecules. In particular, amide A and B bands at 3350 cm^−1^ and 3087 cm^−1^, respectively, are mainly associated with the stretching vibrations of N–H groups. The central portion of both spectra is dominated by amide bands, in particular, amide I band around 1650 cm^−1^ (stretching vibrations of peptide C = O groups), amide II (around 1550 cm^−1^, N–H bending vibrations coupled to C–N stretching vibrations), and amide III (around 1240 cm^−1^, C–N stretching and N–H bending vibrations of amide linkages, plus wagging vibrations of CH_2_ groups in the glycine backbone and proline side chains) [33]. No major shape differences or shift of the position of main bands were detected in the P_PRPE spectrum as compared to P_CTRL. In particular, the amide I band related to the collagen triple helix maintained the same position and intensity, in agreement with existing literature reports [33,34], suggesting that the collagen triple helix is preserved on crosslinking. Further support to this hypothesis is provided by the evaluation of the IR absorption ratios of amide III to adsorption at 1450 cm^−1^, also considered a marker of the preservation of integrity of collagen triple helixes [33]. In both cases the ratio is close to 1, as expected for an integer triple helix. Altogether, surveys of FTIR spectra suggest that interaction of P_CTRL with PRPE to produce P_PRPE does not destroy the backbone structure of collagen.

**Figure 6:**
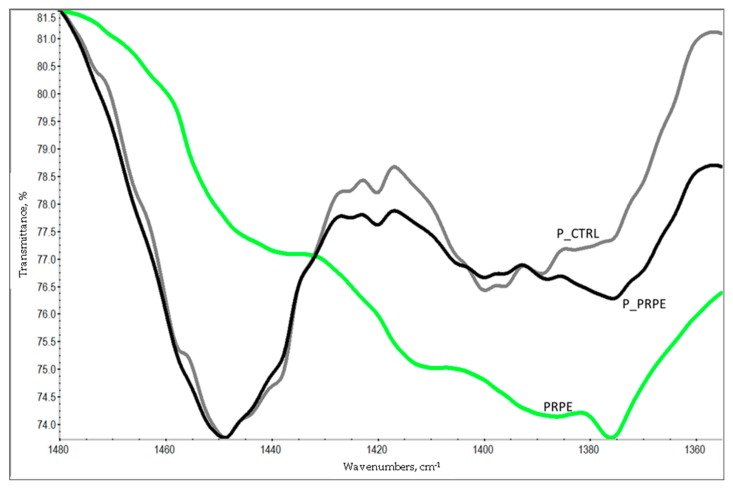
ATR-IR of P_CTRL and P_PRPE and PRPE between 1480 and 1350 cm^−1^.

A further point of interest in the analysis of the ATR-IR spectrum of P_PRPE is the detection of bands due to the PRPE. No significant contribution from procyanidin to the FTIR spectrum of procyanidin crosslinked collagen was reported by He and coworkers [33]. This is reasonable, considering the very strong adsorption of amide groups of protein molecules and the high collagen/polyphenol ratio in the crosslinked film. In the present case, we investigated the possible contribution of PRPE IR adsorption by first obtaining the ATR-IR spectrum of lyophilized PRPE. As expected, it shows very strong adsorption due to hydrogen-bonded hydroxyls at high wavenumbers (3000–2500 cm^−1^) and a very complex pattern in the fingerprint (about 1500 to 500 cm^−1^) region. Detailed comparison of spectra in this area shows interesting features, as reported in Figure 6. In particular, the P_PRPE spectrum shows a weak but definite adsorption at about 1375 cm^−1^, that is not displayed in the P_CTRL spectrum. Interestingly, lyophilized PRPE shows significant adsorption at the same wavenumber, as clearly displayed in Figure 6. It is thus safe to conclude that the different adsorption pattern of P_PRPE vs. P_CTRL in the 1370–1390 cm^−1^ region is due to PRPE “showing-up”. This happens because of the combination in the same small spectral window of the adsorption from protein which is not very strong, and the intense adsorption from PRPE.

### 2.5. Degradation Study

The degradation rate of pericardium treated with PRPE has been tested in a phosphate buffer saline medium and in a solution with a high concentration of collagenase enzymes.

**Figure 7:**
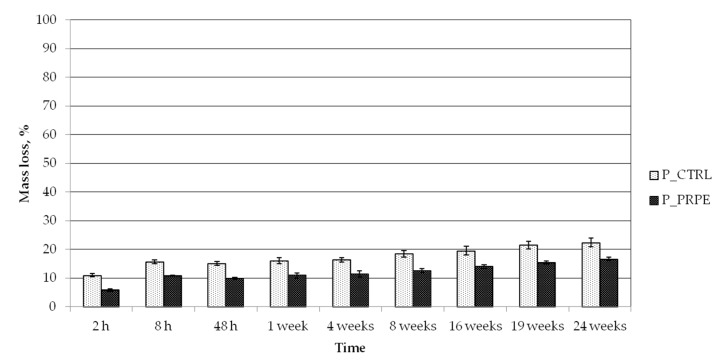
Degradation of P_CTRL (pericardium control) and P_PRPE (pericardium treated with polyphenols), in phosphate buffered saline (PBS) for 24 weeks (6 months).

In the former case, a linear and low degradation rate for both samples was detected. After six months, the decrease of the P_CTRL mass was 22.4% ± 1.5% and P_PRPE lost 16.7% ± 1.6% (Figure 7). The presence of PRPE in the collagen matrix reduces the degradation of the pericardium membrane in PBS solution.

**Figure 8:**
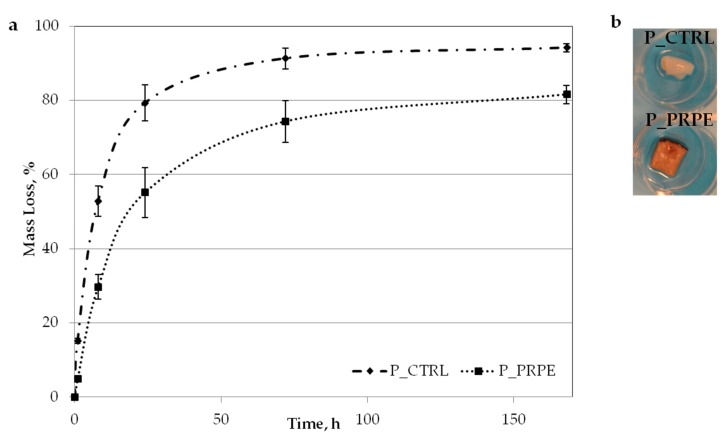
(**a**) Degradation rate of P_CTRL and P_PRPE in collagenase 1 mg/mL over a one week time range. (**b**) Pictures of P_CTRL and P_PRPE after soaking for one week in a solution of 1 mg/mL of collagenase

Samples were further tested in 1 mg/mL collagenase solution to evaluate the effects of the crosslinking procedure on the resistance to enzymatic degradation. We stressed the material by using a high concentration of collagenase enzyme compared to what has been found in vivo in different categories of peri-implant vertical bone loss (around 16 ng/mL for collagenase-3 and around 2021 ng/mL for collagenase-2) [46]. Results are reported in Figure 8.

P_PRPE membrane showed a slow degradation rate and a lower mass loss compared to the membrane non-treated with PRPE (P_CTRL). In particular, after one week the mass loss of P_PRPE and P_CTRL was respectively 81.5% and 94.1%. If we consider that the concentration normally found in vivo is 1000× lower than the one we used in this experiment [46], it is possible to hypothesize that the P_PRPE membrane may have a degradation duration of more than six months.

### 2.6. Pph Release from P_PRPE Membrane

The release study was conducted in PBS solution for 24 h. Each P_PRPE (around 50 mg) was placed in 1 mL of PBS and then the phenolic content and the antioxidant power of the released solution were measured (Table 3). After 24 h, 0.2 mg of GAE and an antioxidant power of 3.2% were detected.

**Figure 9:**
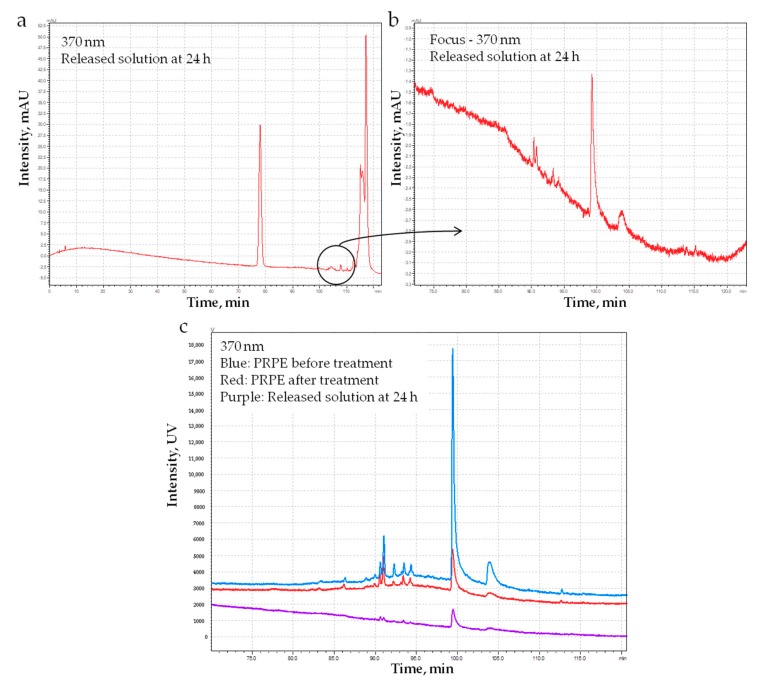
(**a**) HPLC analysis of the released solution after 24 h at 370 nm and (**b**) focus on the peak of quercetin. (**c**) Comparison among the chromatograms of the PRPE before and after the treatment of the pericardium membrane and the chromatogram of the released solution at 24 h.

HPLC analysis of release solutions was also performed (Figure 9). Interestingly, chromatograms detect released quercetin that, as previously shown in Figure 3, is incorporated in high amounts in the collagen network during P_PRPE preparation.

### 2.7. Mechanical Characterization

#### 2.7.1. Tensile Test

**Figure 10:**
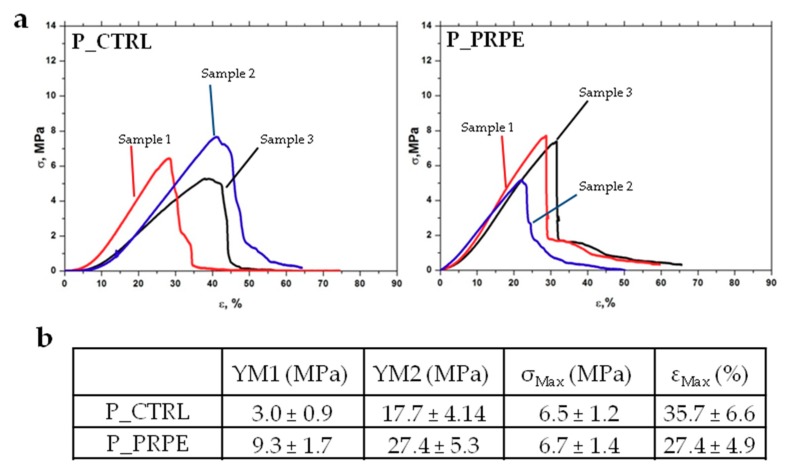
Tensile test in hydrate conditions for P_CTRL and P_PRPE membranes. (**a**) Stress–strain curves for P_CTRL and P_PRPE membranes; three replicates for each sample. (**b**) Mechanical parameters calculated from the stress–strain curves for both membranes.

The tensile test was conducted on P_CTRL and P_PREP in hydrate conditions (30 min of soaking in PBS). Three samples for each type were tested and the results are reported in Figure 10. Usually hydrate samples show stress–strain curves that can be divided into three regions: The first region has a low resistance due to the alignment of the collagen fibers that are in the first step in a rippled structure (region I); then in the second region, there is a linear trend, due to the alignment of the collagen fiber to the stress direction (region II); the resistance is due to the inter and intrafibrillar bonds; in region three, plastic deformation occurs, and rupture of inter and intramolecular bonds leads to the failure of the sample (region III).

In the present case, we calculated two Young’s moduli: one from region I and one from region II. As shown in Figure 10a, region I for P_PRPE membrane is shorter than the corresponding P_CTRL. This is probably due to the crosslinking of PRPE, which reduces swelling and water uptake ability of the membrane. The Young modulus of the first zone, is three-fold higher for P_PRPE compared to P_CTRL (9.3 ± 1.7 MPa and 3.0 ± 0.9 MPa, respectively) (Figure 10b). Improvement of mechanical tensile properties, due to the crosslinking with PRPE is also confirmed by the increase in the Young modulus calculated in region II (YM2), which is 27.4 ± 5.5 MPa for P_PRPE and 17.7 ± 4.14 MPa for P_CTRL. Obviously, also the maximum elongation is reduced, from 35.7 ± 6.6% for P_CTRL to 27.4 ± 4.9% for P_PRPE.

#### 2.7.2. Tear Test

**Figure 11:**
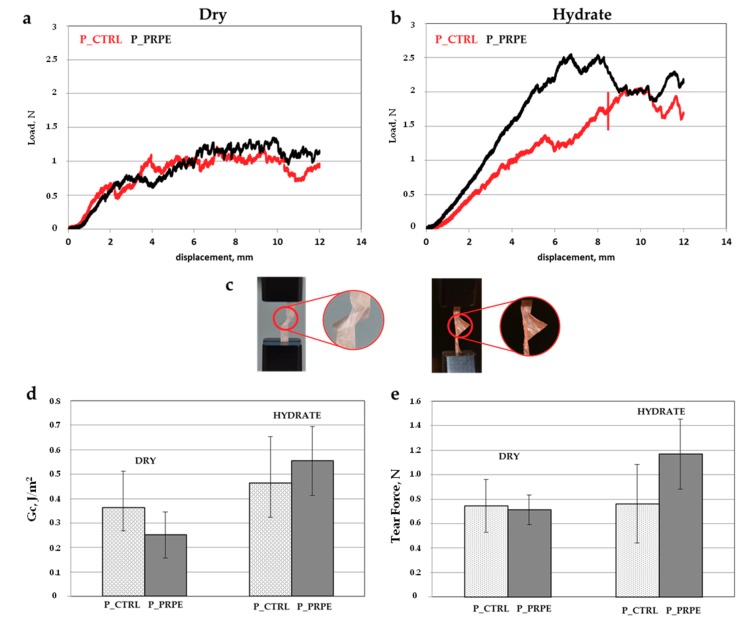
Tear test of P_CTRL and P_PREP membranes. (**a**,**b**) displacement-force curve in dry and hydrate conditions for P_CTRL and P_PRPE membrane. (**c**) Picture of the effect on P_CTRL (left) and P_PRPE (right) due to the tearing stress. (**d**) Histogram of the energy required to tear in both dry and hydrate conditions for P_CTRL and P_PRPE. (**e**) Maximum tear load in hydrate and dry conditions for P_CTRL and P_PRPE membranes.

The tensile test is an easy way to compare different materials, however it is not the most common mechanical stress to which membranes for periodontal regeneration are usually subjected. Material failure under tensile load is quite difficult in the guided bone regeneration/guided tissue regeneration GBR/GTR application [47,48]. Failure of this type of materials is much more probably provoked by tearing during placement. The tear tests give a better comparison among different materials as they provide information on the energy or force required to propagate a tear through the material. The tear test was performed in dry and hydrate conditions, and initiated with a 7 mm initial cut, propagation was monitored until a maximum displacement of 10 mm at the rate of 1 mm/min; obtained results are reported in Figure 11. Tear load (Figure 11a) is reported as a function of displacement, and the curves are characterized by a scattered shape, in particular in dry conditions, which is a behavior typical of fibrous materials [49].

In this study a trouser tear test was performed and the two legs are fixed in the longitudinal fixation grip. With this set up, the samples show a lower tear load in dry condition, that is the condition in which the fibers are more rigid and the applied force easily breaks the fibers instead of stretching them. In hydrate condition, the situation is slightly different, the scattering is reduced and the tear load increases. However, no significant difference between P_CTRL and P_PRPE in both dry and hydrate condition was detected. During the experiment, both membranes (P_CTRL and P_PRPE) arrange the fiber in the direction of the stress and reduce the section, increasing the resistance to the tear, blocking it. In fact, both membranes do not reach the break (Figure 11c). The crosslinking of the P_PRPE is slightly evident in hydrate condition, where the tear force of the P_PRPE is 1.17 N while it is 0.76 N for P_CTRL (Figure 11e).

The tear stress can be divided in two steps: in the first part fibers start to re-arrange during tearing along the direction of the stress and the principal reaction force is due to the interfibrillar bond. In the second step the ensuing orientation increases tear strength. In this second part the intrafibrillar bond became more relevant and differently to tensile test, during tear test membranes are subjected to shear stresses and the mobility of the fiber could obstruct the crack propagation [50]. The energy calculated and reported in Figure 11d is the energy required to re-arrange the material and to avoid the break. In dry condition, the energy is lower than in the hydrate condition: since the deformability of the membrane influences the mobility of the fiber, the rigidity of both membranes in dry condition makes easier the crack propagation compared to the hydrate condition.

#### 2.7.3. Suture Retention Strength Test

GTR/GBR application is usually a low load bearing application, for which a suture in order to fix the material is rarely necessary, but in some cases, it could be necessary. The measure of the suture retention strength was conducted following the ANSI/AAMI/ISO 7198:2016 (revision 2016, Association for the Advancement of Medical Instrumentation (AAMI), Arlington, VA, USA) “Cardiovascular implants and extracorporeal systems—Vascular prostheses—Tubular vascular grafts and vascular patches” procedure, and setup is shown in Figure 13. As suture a Polypropylene thread USP 3/0 was used. The test was performed in hydrate condition, to mimic the clinical practice.

**Figure 12:**
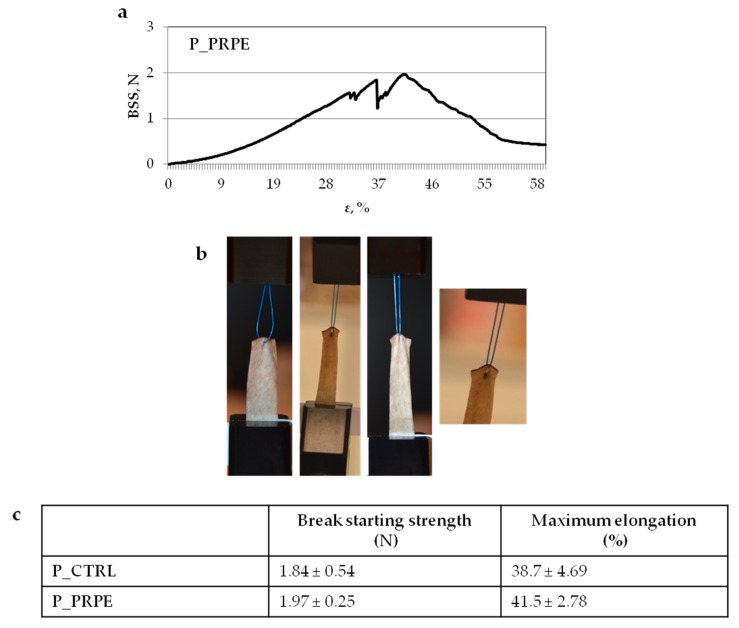
Suture retention strength test using Polypropylene monofilament USP 3/0 surgical suture. (**a**) stress–strain curve for P_PRPE sample. (**b**) Picture of the sequence during the suture retention test for P_PRPE membrane. (**c**) Mechanical parameters calculated from the stress–strain curve of the suture retention strength test.

The measured break retention strength and the related elongation are reported in Figure 12. A trend towards the increase of the retention strength and the elongation is detected for P_PRPE compared to P_CTRL, on the average. The crosslinking of the fiber results in an increase in the ability of the pericardium to contrast the propagation of the crack. In fact, the break starting strength is 1.97 ± 0.25 N for P_PRPE and 1.84 ± 0.54 N for P_CTRL. The maximum elongation is also higher, which is probably due to the ability to resist against the tensile suture stress with the intrafibrillar bond which starts to break gradually and allows the membrane to elongate until the catastrophic crack. However, no significant differences have been detected between the two materials, this is due to the fact that the notch generated at the moment of needle insertion is what dominates specimen rupture [51].

## 3. Discussion

Membranes for GTR are an important tool in today’s dental surgery. While commercially available membranes provide satisfactory results, improvements are still investigated in terms of durability and ancillary therapeutic actions.

Following increasing research interest on the potential role of Pph in medical materials’ engineering and in dental materials’ applications [32], in this work we investigated the effect of a readily available Pph source (pomace extracts) on clinically relevant properties of a collagen membrane, obtained from porcine pericardium. PRPE was obtained from Nebbiolo cultivar, recognized as one of the grapes with the highest amount of tannins (flavanols monomers and polymerized) and flavones which have been used as effective crosslinking agents with collagen [35,36,52]. The main idea behind the treatment of the pericardium membrane with PRPE is to stabilize the membrane with a natural, non-toxic component, in order to match the need for periodontal regeneration through inhibiting the enzymatic scissoring and to also endow the membrane with an ancillary therapeutic effect due to the released molecules in situ. In particular, the well-known anti-inflammatory properties of polyphenols and their influence on the molecular pathway involved in bone regeneration [42,53] can be of help in case of periodontitis or perimplantitis [52].

Collagen-based materials are extensively used for the manufacture of biodegradable GTR membranes due to the biological activities of collagen, its low immunogenicity, and stimulation of the coagulum. However, the biodegradable barrier provides limited control over the length of application due to the disintegration process of the enzymes. The degradation rate is an important aspect of the clinical function and the loss of the structural integrity of the membrane is a major problem with these types of bioabsorbable devices. For a successful bone regeneration, the bone defect needs to be separated from the soft tissue for at least 16–24 weeks [54,55]. In order to control the degradation time of the collagen membranes, the most common procedures are the crosslinking methods (e.g., ultraviolet radiation, glutaraldehyde, diphenyl-phosporyl-azide, hexamethylenediisocyanate) [56,57,58,59]. These methods decrease the degradation rate, but on the other hand, some of the crosslinking techniques make the collagen-based membranes cytotoxic, thus reducing cells’ attachment and proliferation. Furthermore, the classical crosslinking approach increases the resistance of the material by bonding the chain but without any active effect of the enzyme such as the collagenase. By analyzing the sulcus fluids from patients with a different amount of bone loss, concentrations of collagenase-2 and -3, in the order of ng/mL, were found. In our study we decided to stress the membrane, by using a 1000× higher collagenase concentration, in order to analyze the effect of Pph even in the worst case, thus also reducing the time analysis and the material required.

Obtained results show that Pph from PRPE are indeed incorporated into the collagen network, and part of them can be released in situ. In the present work, we did not try to define a release rate or profile, thus limiting our analysis to the evaluation of the reversibility of the collagen–Pph interaction. As a first result of the Pph treatment, the degradation rate of the membrane was decreased, either through chemical crosslinking of the collagen chain or inhibiting the action of collagenase [60] (Figure 8). Pph interact with collagen fibers by binding to them and by inhibiting collagenase activity. They bind to collagen through different mechanisms, such as hydrogen bonding mechanisms with both an arginine side chain and an aspartic acid peptide backbone, stabilized through hydrophobic interactions [61,62,63]. The polyphenol tannic acid, with its multiple phenolic residues, has a high affinity for the hydrophobic sites present in the collagen, thus preventing collagenase action on those cleavage areas. The PRPE used in the present work contain a high amount of quercetin which is, as shown in Figure 3 and Figure 9, largely uptaken and released by the membrane. It has been reported that the flavones, and in particular quercetin, are some of the most effective inhibitors of collagenase [64]. The pericardium also contains elastin, and it has been shown that Pph, in particular, proanthocyanidin molecules, can prevent enzyme activity by binding to amino acid residues in a way that prevents the disruption of the polypeptide chain [65,66]. The results obtained in the degradation test are clinically important, since they show that by using even the highest amount of enzymes, the Pph has an inhibitory effect which could be amplified through lowering the enzyme concentration.

Local delivery of Pph, as shown in Figure 9, is important because the role of polyphenols in bone regeneration has been widely demonstrated [41]. Furthermore, polyphenols and in particular, quercetin, exert their anti-resorbing action by regulating inflammatory cytokines responsible for bone resorption and subsequently degenerative bone diseases [67].

Present results confirm the effect of PRPE on mechanical properties of GTR membranes from porcine pericardium. Pericardium is a fibro-serous sac surrounding the mammalian heart. It is mostly composed of fibrous connective tissue and, thanks to its exceptional handling characteristics and uniform suture retention, has been widely used in a variety of cardiovascular applications. The native structure of the pericardium is composed of three layers: (1) the serosa, the inner thin layer consisting of mesothelial cells; (2) the fibrosa, a thicker layer composed of diversely oriented fibers of collagen and elastin; (3) the epipericardial connective tissue layer. When the pericardium is processed the fibrosa layer is maintained, composed by the woven fibers of collagen and elastin. This fibrous layer of porcine pericardium possesses a greater uniformity and the biomechanical properties are directly related to the distribution and orientation of the collagen fibers. The structure of the porcine pericardium, characterized by the presence of a network of fine multidirectional collagen fibers arranged in layers composing the fibrous layer, is in accordance with its function. Pericardium is subject to diastolic force and the orientation of the fibers guarantees the restraint of this movement. Owing to its properties, porcine pericardium has found some applications in dentistry, in particular, it has been used in the following fields: alveolar ridge augmentation [68], guided bone/tissue regeneration [69], root coverage, and treatment of dehiscence defects [70].

Contrary to cardiovascular applications, in which the mechanical stress plays a fundamental role, in dental application, in particular GTR, it is important to find a crosslinking agent to protect against enzymatic degradation without influencing the mechanical properties. It has been reported that the use of glutaraldehyde as a crosslinking agent makes the pericardium stiff and contracted [71]; this should be avoided in the dental field, where handling and easiness in placing the membrane in situ is mandatory.

Present data show excellent mechanical properties of P_PRPE. Tensile data indicate that the crosslinking process makes the membrane much more manageable, combining an excellent mechanical stiffness with good elasticity that improves handling in clinical use.

Results obtained in the tear test are comparable with the values reported in literature for commercially available membranes, which showed a range of value between 0.6 to 2 N for the tear force [49]. The complexity of the pericardium network does not allow for detection of significant differences between P_CTRL and P_PRPE since the hydrogen bonds created by polyphenols with the fibers appear to have less influence on the tear stress than in the tensile test. However, from a clinical point of view it is really important that the properties of the membrane are not negatively influenced by the treatment process with polyphenols.

The interaction between one suture point and the material is usually assessed to quantify the suture retention strength. However, in accordance with what is already reported in literature, this value strongly depends on the geometry of the sample, and furthermore it corresponds to catastrophic failure. For these reasons, we decided to report the break retention strength, which is more conservative, but it does not depend on the size of the suture thread and is practically independent from the test geometry. The use of a needle to pass the suture through the samples induces a notch in the tested membrane, hence the crack propagates. In this context, the role of wire is just to pull the notch surfaces apart, until a critical limit is reached. Normally, this is a common test in the cardiovascular field, and in that field novel grafts are typically characterized based on their capability to exceed a 2.0 N threshold in terms of retention strength.

## 4. Materials and Methods

### 4.1. Materials

All chemicals were analytical-reagent grade. Ultra-pure (MilliQ) water was used for the preparation of aqueous solutions. All chemicals, that is acetone, acetic acid, Folin–Ciocalteu reagent, 2,2-Diphenyl-1-picrylhydrazyl, sodium carbonate, sodium bisulfite, gallic acid, quercetin, rutin, caffeic acid, p-cumaric acid, and malvidin-3-glucoside were purchased from Sigma-Aldrich (St. Louis, MO, USA). Red grape pomace were purchased from a local winery producer (grape Nebbiolo, from Producers of Barabresco, CN, Italy).

### 4.2. Preparation of Polyphenol-Rich Pomace Extracts (PRPE)

Grape pomace were received dry from the producer and stored at −20 °C under vacuum until the beginning of the extraction process. To make it suitable for extraction, pomace were first washed with acidified water, dried in a circulating-air oven (UN110, Memmert GmbH, Schwabach, Germany) (37 ± 5 °C), and grinded in a bladed mill (GM 200, Retsch GmbH, Haan, Germany). The milled grape pomace (300 g) were extracted in 2000 mL of 50:50 acetone:water (*v*/*v*) by using an automatic extractor (Micro C, TIMATIC USA, Oakland, CA, USA). The extraction cycle is fully automatic and alternates a dynamic phase, performed at a programmed pressure, and a static phase in which a forced percolation is generated, which, thanks to the programmable recirculation, ensures a continuous flow of solvent to the interior of the plant matrix, thus avoiding over-saturation. Next, the extracted solution was concentrated under reduced pressure in a rotary evaporator and maintained in a fridge between 2–4 °C.

### 4.3. Preparation of PRPE Crosslinked Porcine Pericardium Membranes

Pericardium form porcine origin, supplied by a local abattoir, was cleaned from the residual fat and decellularized by using the procedures reported in the article of Courtman et al., based on a combination of hypotonic buffer, Triton-X100, and nucleases [72]. Then, the specimens of whole pericardium were cut in rectangular shape samples (40 × 40 mm) and soaked in distilled water (P_CTRL) or PRPE (P_PRPE) for 2 h, washed with distilled water 3 times, and freeze-dried overnight.

### 4.4. HPLC Analysis

PRPE from Nebbiolo was characterized by using high performance liquid chromatography (HPLC, LC 2010 AHT equipped with Diode array SPD-M10AVP, Shimadzu corporation, Kyoto, Japan) technique. PRPE was filtered through 0.2 µm cellulose acetate filters (Target2TM, Thermo Scientific, Waltham, MA, USA) and analyzed using a C8 Luna column (150 × 4.6 mm; 5 µm particle size) from Phenomenex (Torrance, CA, USA) and operated at 25 °C. The mobile phases consisted of 2% (*v*/*v*) acetic acid in water, Mobile Phase A (MPA) and 0.5% acetic acid in water and acetonitrile (50:50 *v*/*v*), Mobile Phase B (MPB), by using the gradient program reported in Table 4, at a flow rate of 0.8 mL/min and a total run time of 123 min [73].

The injection volume was 10 µL and the diode array operated in the wavelength range from 200 to 600 nm. Main Pph were identified through comparison with reference compounds. The quantitation of individual Pph was performed by using calibration curves of the corresponding reference compounds. Gallic acid (280 nm), quercetin (370 nm), rutin (355 nm), and malvidin-3-glucoside (520 nm) were dissolved in ethanol: water solution at the concentration of 1, 5, 10, 50 100, 150, and 200 µg/mL and analyzed with the same method reported above. The quantitation was performed by applying the standard calibration curve.

### 4.5. Phenolic Content of PRPE

The initial phenolic content of Nebbiolo PRPE was evaluated by using the Folin–Ciocalteu method (FC). The extract was transferred in a 25 mL volumetric flask (Durnan Group GmbH, Mainz, Germany) and diluted 1:50 with distilled water. Then, 0.5 g of Folin–Ciocalteu reagent was added and mixed for 5 min and 1.5 g of 20% anhydrous sodium carbonate (*w*/*v*) solution was added. After 2 h, the absorbance was measured at 765 nm, by using water as the compensation liquid and a quartz cell (PG Instruments Limited, Leicestershire, UK) (10 mm path length) in a UV–Vis spectrophotometer (T80+, PG Instruments Limited, Leicestershire, UK). The absorbance value was used to calculate the concentration of polyphenols by using a calibration curve obtained with gallic acid. The results are expressed as mg/mL of gallic acid equivalents (GAE).

Calibration curve: 10 mg of gallic acid was diluted in 10 mL of water to obtain 1 mg/mL of stock solution. Aliquots of stock solution were transferred in a 25 mL volumetric flask and diluted in water to the final concentrations of 0.05 mg/mL, 0.025 mg/mL, 0.01 mg/mL, and 0.005 mg/mL. Each standard solution was prepared according to the procedure described above for the PRPE; the absorbance was measured under the same conditions as for PRPE.

### 4.6. Antioxidant Power of PRPE

The antioxidant power of PRPE (1 mg/mL of GAE) and of Pph released from P_PRPE was evaluated through the widely used DPPH (2,2-Diphenyl-1-picrylhydrazyl) method described by Brand-Williams et al. [74]. Using a colorimetric approach, this test measures the ability of the test solution to scavenge the DPPH radical. Shortly, an aliquot of 40 µL of PRPE was added to a volume of 1600 µL of water: ethanol 50:50 (*v*/*v*) solution. Separately a DPPH solution (0.1 mg/mL *w*/*v*) in ethanol was prepared and 2 mL of this solution was added at the reaction mixture. Then the solution was shaken and incubated for 30 min at room temperature in the dark; the absorbance was recorded at 525 nm. Blank solution was constituted by a solution of water: ethanol instead of PRPE. The percentage inhibition of the DPPH radical by the samples was calculated using the following equation:

(1) $$\%\;\mathrm{Reduction}=\left(\frac{A_{0}-{\;A}_{1}}{A_{0}}\right)\times 100$$

where A_0_ is the absorbance of control sample and A_1_ is the absorbance of the test sample.

### 4.7. Determination of Anthocyanins in PRPE

The method of Ribéreau-Gayon and Stonestreet was used to determine the concentration of proanthocyanidins in PRPE [75]. It exploits the transformation of anthocyanin into colorless derivatives under the action of certain reagents such as bisulfite ions. Since anthocyanin absorbance occurs around 520 nm, its decrease after the addition of excess bisulfite ions is proportional to the anthocyanin content.

Briefly, solution A was prepared in a test tube of 50 mL as follows: mix 1 mL of PRPE with 1 mL of acidified ethanol solution (0.1% *v*/*v* hydrochloric acid, HCL, Sigma-Aldrich, St. Louis, MO, USA) and 20 mL of HCl solution (2% of HCL in distilled water). Then, in a 25 mL test tube, 5 mL of solution A were mixed with 2 mL of Milli Q water (solution B) and in another 25 mL test tube, 5 mL of solution A were mixed with 2 mL of sodium bisulfite solution (concentration of 150 g/L) (solution C).

Through an UV–Vis spectrophotometer the absorbance at 520 nm was recorded for both solutions prepared, and the absorbance variation was used to calculate the amount of anthocyanins contained in the PRPE, by using a calibration curve made through the standard anthocyanin malvidin-3-glucoside. Results are expressed as µg/mL of malvidin-3-glucoside equivalent.

### 4.8. Scanning Electron Microscopy

Scanning electron microscopy analysis was performed to analyze the morphology of membranes. Samples were mounted on the aluminum stubs and sputtered with gold at 15 mA for 2 min using Agar Sputter Coater. The morphology of samples was captured by using a scanning electron microscope (SEM) EVO MA 10 system (Zeiss, Oberkochen, Germany). Relevant image acquisition data are reported in the figure data bar (Figure 4).

### 4.9. ATR-IR

The composition changes at the surface level of the pericardium membrane (1 to 2 µm depth) were analyzed through attenuated total reflectance infrared spectroscopy analysis (ATR-IR). ATR-IR was performed by using a Nicolet iS 10 ATR-IR spectrometer, produced by Thermo Scientific (Waltham, MA, USA) and equipped with a diamond crystal. Samples to be analyzed were placed on the crystal and kept in place by the specific crimping tool. Experimental setup was conducted by acquisition of 32 scans in the range of 500–4000 cm^−1^, with a resolution of 4 cm^−1^.

### 4.10. Degradation Study

The stability of the samples was analyzed in PBS at 37 °C. The samples, five for each type, were cut in a cubic shape (10 mm in diameter and 2 mm in height; or 1 × 1 × 1 cm^3^) and immersed in PBS or in a solution of collagenase at the high concentration of 1 mg/mL, after recording their initial weight (W_0_). During the degradation study, samples were taken out at specific time intervals, freeze-dried, and weighted (W_f_). The mass loss percentage was calculated by using the following equation:

(2) $$\%\;\mathrm{Mass}\;\mathrm{loss}\;=\left(\frac{W_{0}-{\;W}_{f}}{W_{0}}\right)\;\times \;100$$

### 4.11. Release Study

Pph release study was performed by using the Folin–Ciocalteu method to quantify the phenolic content in the release media and the DPPH assay was used to quantify the antioxidant power, as described in the previous section. HPLC analysis was performed on the release solution according to the method described in Section 4.4. Five specimens for each sample were immersed in PBS solution (1 mL of PBS for 50 mg of pericardium) at 37 °C for 24 h. At this time point, samples were taken out and the released solution was analyzed.

### 4.12. Mechanical Characterization

Mechanical characterization was performed by using a Bose ElectroForce 5500 (TA Instruments, New Castle, DE, USA) equipped with 100 N load cell.

#### 4.12.1. Tensile Test

Membranes were cut in a rectangular shape with 20 mm gauge length and 2 mm height. Mechanical properties were tested in hydrated conditions, after soaking in PBS for 30 min. For the uniaxial tensile test, samples were stretched until failure (or until the maximum displacement of the testing machine) at the crosshead of 0.2 mm/s. Force-displacement curves, obtained from the machine, were converted to stress–strain curves. The stress (σ, MPa) was obtained by dividing the applied force (N) with cross section area (mm^2^), and strain was obtained from the displacement using ((*L*-*L*_0_) × 100/(*L*_0_)), where *L*_0_ was the initial gauge length and *L* was instantaneous gauge length. Young’s modulus was calculated in the linear stress–strain region by fitting a straight line.

#### 4.12.2. Tear Stress Test

The tear test provides information on the force required to propagate a tear through the material. Three specimens were cut with a nominal area of 5 × 15 mm^2^ and the test was initiated with 7 mm long central cut. Tear propagation was monitored as a function of the vertical displacement at the constant rate of 1 mm/min up to a maximum displacement of 10 mm. The tear test was performed for both samples with and without PRPE and in two conditions, dry and hydrated (PBS for 30 min). Tear force and energy required to tear were calculated. Tear force was the maximum force registered between 4 and 6 mm of displacement. The energy required to tear was calculated using the following equation (Equation (3)):

(3) $$G_{c}=\frac{1}{t\times \;L_{bulk}}\int Fd\Delta$$

where *F* is the load, Δ is the displacement, *t* is the thickness, and *L_bulk_* the length of the tear.

#### 4.12.3. Suture Retention Strength

**Figure 13:**
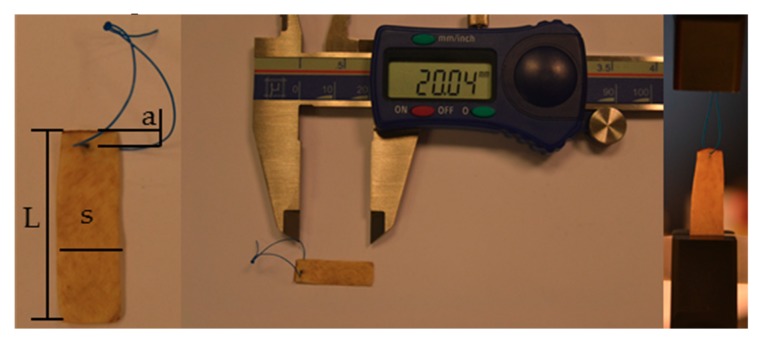
Suture retention strength sample preparation.

Suture retention tests were performed on rectangular specimens clamped at the edge located opposite to the suture. Protocols were adopted from the methods described within the ANSI/AAMI/ISO 7198:2016 Cardiovascular implants and extracorporeal systems—Vascular prostheses—Tubular vascular grafts and vascular patches [76]. A suture thread was passed through the sample by a needle, then closed into a loop by means of multiple knots, and finally closed in the others clamps (Figure 13). The suture bite was centered with respect to the specimen width (s) and its distance from the clamp was *L*_0_ = *L*−a (see Figure 13). The dimensions adopted for the test were: *L* = 20 mm, s= 10 mm and in accordance with the standard, samples were sutured at a minimum distance of 2 mm (Figure 13) from the sample’s free end (see Figure 13). A preload of 0.1 N on the suture-pericardium interface was applied, then a displacement speed of 1 mm/sec was set. Following the preload, a ramp-to-failure test was executed by utilizing WinTest® 7.1 software’s waveform setup utility. In accordance with the article of Pensalfini et al., the corresponding force named break starting strength (BSS) [51] was recorded. As described in [47] BSS does not depend on the geometry of the sample and it is more conservative than suture retention strength value which corresponds to catastrophic failure.

## 5. Conclusions

The present work highlights the effect of polyphenol-rich extract from grape pomace on the properties of porcine pericardium. In particular, results show that the interaction of PRPE with collagen inhibits the enzymatic breakdown of the membrane structure and delays degradation. The use of PRPE as a natural crosslinker of porcine pericardium, allows for a novel barrier membrane endowed with improved mechanical properties to be produced. Further studies are needed to also understand the biological effect of polyphenols incorporated and released by the membrane tissue and their relevant clinical implications.

## Figures and Tables

**Table 1:** Phenolic content (gallic acid equivalent (GAE) mg/mL), antioxidant power (reduction (%) of DPPH radical) and anthocyanin content of Nebbiolo PRPE.

PRPE	GAE (g/mL)	Reduction (%)	Anthocyanins (µg/mL)
Nebbiolo	3.44	44.3	74.4

**Table 2:** Quantification of four different polyphenol molecules through HPLC analysis.

PRPE	Gallic Acid (mg/L)	Quercetin (mg/L)	Rutin (mg/L)	Malvidin-3-Glucoside (mg/L)
**Nebbiolo**	19.43	66.46	38.45	3.76

**Table 3:** Phenolic content and antioxidant P_PRPE membrane power of the release solution.

Phenolic Content (mg/mL GAE)	Antioxidant Power (%)
0.2 ± 0.12	3.2 ± 0.6

**Table 4:** Details of the HPLC gradient method used for PRPE analysis.

Time (min)	MPA (%)	MPB (%)
0–35	100 → 95	0 → 5
35–80	95 → 80	5 → 20
80–110	80 → 0	20 → 100
110–113	0	100
113–123	0 → 100	100 → 0
